# Applied Motor Noise Affects Specific Learning Mechanisms during Short-Term Adaptation to Novel Movement Dynamics

**DOI:** 10.1523/ENEURO.0100-24.2024

**Published:** 2025-01-10

**Authors:** Katherine Foray, Weiwei Zhou, Justin Fitzgerald, Pierre G. Gianferrara, Wilsaan M. Joiner

**Affiliations:** ^1^Departments of Neurobiology, Physiology and Behavior, University of California, Davis, Davis, California 95616; ^2^Neurology, University of California, Davis, Davis, California 95616

## Abstract

Short-term motor adaptation to novel movement dynamics has been shown to involve at least two concurrent learning processes: a slow process that responds weakly to error but retains information well and a fast process that responds strongly to error but has poor retention. This modeling framework can explain several properties of motion-dependent motor adaptation (e.g., 24 h retention). An important assumption of this computational framework is that learning is only based on the experienced movement error, and the effect of noise (either internally generated or externally applied) is not considered. We examined the respective error sensitivity by quantifying adaptation in three subject groups distinguished by the noise added to the motion-dependent perturbation. We assessed the feedforward adaptive changes in motor output and examined the adaptation rate, retention, and decay of learning. Applying a two-state modeling framework showed that the applied noise during training mainly affected the fast learning process, with the slow process largely unaffected; participants in the higher noise groups demonstrated a reduced force profile following training, but the decay rate across groups was similar, suggesting that the slow process was unimpaired across conditions. Collectively, our results provide evidence that noise significantly decreases motor adaptation, but this reduction may be due to its influence over specific learning mechanisms. Importantly, this may have implications for how the motor system compensates for random fluctuations, especially when affected by brain disorders that result in movement tremor (e.g., essential tremor).

## Significance Statement

Short-term motor adaptation to novel movement dynamics has been shown to involve at least two concurrent learning processes: a slow process that responds weakly to error but retains information well and a fast process that responds strongly to error but has poor retention. This computational framework assumes that learning is only based on the movement error, and the effect of noise is not considered. We found that as the magnitude of externally generated noise increased, the overall learning rate decreased, which could be explained specifically by impairments to the fast learning process. The applied motor noise had little effect on the retention and decay of adaptation—aspects that mainly involve the slow learning process.

## Introduction

Motor learning is a type of experience-dependent learning that involves changes in behavioral output to achieve a desired outcome (Wolpert et al., 2001, 2011; Halsband and Lange, 2006; Krakauer et al., 2019). The nervous system integrates different sources of information (e.g., externally generated sensory feedback, internally generated predictive signals, etc.) to determine the appropriate motor output to compensate for experienced errors (Mazzoni and Krakauer, 2006; Wolpert et al., 2011; Krakauer et al., 2019). Motor adaptation, a form of short-term motor learning, can occur on a trial-by-trial basis (i.e., error-based learning) to recalibrate motor output to reduce sensory prediction errors (Synofzik et al., 2006, 2008; Berniker and Körding, 2008; Shadmehr et al., 2010; Izawa and Shadmehr, 2011; Wolpert et al., 2011; Butcher et al., 2017).

Motor adaptation of arm reaching movements (Shadmehr and Mussa-Ivaldi, 1994) is typically studied using a robotic manipulandum. Participants hold the handle of the robot and are asked to make reaching movements between targets in the presence of a dynamic, lateral force-field (FF) perturbation (Joiner and Smith, 2008; Mckenna et al., 2017; Alhussein et al., 2019; Zhou et al., 2022). These FF perturbations are usually orthogonal to the direction of the arm reaching motion, and the amount of force is proportional to the motion state of the movement (e.g., movement velocity). During this perturbation, participants must adapt the temporal pattern of force to counteract the lateral force of the perturbation (Conditt et al., 1997; Conditt and Mussa-Ivaldi, 1999; Sing et al., 2009, 2013; Yousif and Diedrichsen, 2012; Hosseini et al., 2017; Joiner et al., 2017). Interspersed randomly throughout the task, error-clamp (EC) trials restrict movement (in the absence of the perturbation) so that participants are guided toward the target in a straight line while keeping lateral errors very small (Scheidt et al., 2000; Hwang et al., 2006; Smith et al., 2006; Wagner and Smith, 2008; Sing et al., 2009; Wei and Körding, 2010; Wanda et al., 2013). EC trials capture the applied force of the subject and provide a measure of adaptation; the temporal profile of the lateral force produced by subjects is compared with the ideal force pattern based on the measured motion state throughout the movement (e.g., movement velocity).

Retention of motor adaptation of arm reaching movements can be modeled as a multi-rate, gain-independent two-state model developed by Smith et al. (2006). This framework postulates that adaptation is the result of two concurrent learning processes: one process that responds quickly to movement errors but has poor retention (i.e., the fast process) and another that responds slowly to movement errors but retains the learning well from one trial to the next (i.e., the slow process; Smith et al., 2006; Joiner and Smith, 2008; Shadmehr et al., 2010; Wolpert et al., 2011; Albert and Shadmehr, 2018; Sarwary et al., 2018; Coltman et al., 2019). The two-state model has been shown to account for the retention of adaptation following a 24 h period (Joiner and Smith, 2008); at the end of training, the best predictor of retention is the slow learning process's efficiency over time as opposed to the overall adaptation level reflective of learning, as might have been expected. In a further attempt to understand which specific mechanisms during training affects the stability of adaptation, Alhussein et al. (2019) systematically examined the influence of training duration and type of exposure (gradual vs abrupt) on the short-term decay of learning. The authors found that the training duration had the strongest effect on adaptation stability (i.e., the persistence of the adapted motor output over a period of time); the longer training durations resulted in a slower decay of adaptation, independent of the type of exposure. Using the two-state modeling framework, the authors further showed that the slow learning process was best able to predict the stability; the less stable the slow learning process, the faster the adaptation decayed. Thus, the studies above show that the slow learning process plays a substantial role in the long-term memory formation and stability (observed by the time course of decay) of motor adaptation.

One current limitation of the two-state model is its interpretation of the error as a perturbation originating from a single source. That is, the model assumes that the motor error that drives learning has idiosyncratic consistent characteristics. However, error can possess different characteristics, such as source, magnitude, and frequency (Faisal et al., 2008; Wolpert and Landy, 2012). While the slow learning process is most influenced by the duration of training and evolves over time, the fast learning process is characterized by quick responses to movement errors, which are rapid changes in the environment resulting in motor variability (Smith et al., 2006; Alhussein et al., 2019). However, it is unclear how the error sensitivity of the respective processes changes when faced with variability. That is, it is unknown to what extent the learning mechanisms adjust their response when faced with fluctuations in the experienced error. Thus, there is a need to understand the exact relationship between variability in error, or noise, and the subsequent effect on these two learning processes. By understanding the impact that different noise parameters have in adaptation, we will gain greater insight into how variability in the experienced error influences motor learning. This in turn will allow updating the current models of adaptation to reflect the compensation to real-world movement errors.

In addition to the underlying temporal and spatial characteristics, motor noise can be broadly categorized as either internally or externally generated (coming from a source within or separate from the organism; Berniker and Körding, 2008; Faisal et al., 2008). Understanding this influence will assist in assessing the consequences of noise on the motor behavior in certain patient populations. For example, motor noise has been shown to impair the ability to appropriately adapt motor output when either internally generated (McCrea and Eng, 2005; Kronenbuerger et al., 2007; Shill et al., 2009) or externally applied (Therrien et al., 2018). This is particularly relevant to patients with pathological tremor, a type of internally generated noise, who have impaired motor learning (McCrea and Eng, 2005; Kronenbuerger et al., 2007; Shill et al., 2009). Specifically, patients with essential tremor (ET) are known to have a high-frequency tremor (6–12 Hz; Agarwal and Biagioni, 2024) that impairs their ability to adapt (Shill et al., 2009). In a study examining visual sensory feedback noise, higher amounts of externally generated noise during adaptation have been suggested to require compensation from explicit learning strategies (Miyamoto et al., 2020), which have been thought to constitute the fast learning process of the two-state model (McDougle et al., 2015). While these studies provide initial insight into how noise impairs motor adaptation, there has not yet been direct evidence of the specific mechanisms and timescales by which these impairments occur. To better understand the precise aspects of motor learning affected by internally generated noise, especially in disease models, we must first establish how varying characteristics of externally generated noise with the same frequency as essential tremor impacts healthy participants.

The objective of this study was to assess motor adaptation in the presence of additional noise of different magnitudes. By applying the two-state model, we aimed to further understand the impact of externally applied motor noise on different characteristics of motor learning (e.g., adaptation rate, retention, and decay). Through the addition of noise with the same frequency as essential tremor to movement error, we hoped to gain greater insight into real-world representations of movement error and contribute to the current two-state model, which currently interprets error as being nonvariable. We hypothesized that as the magnitude of externally generated noise increased, the motor adaptation rate in response to the novel movement dynamics would decrease, specifically as a result of impairment of the fast learning process. Subsequently, due to a largely intact slow process, the specificity of impairment would result in similar retention and decay rates of adaptation across the different levels of applied noise. Ultimately, a more comprehensive understanding of error sensitivity will provide insight into how the motor system compensates for random fluctuations, especially when affected by brain disorders that result in movement tremor (e.g., essential tremor or Parkinson's disease).

## Materials and Methods

Sixty right-handed participants (15 male, 44 female, and 1 unknown; aged between 18 and 30 years) without known neurological impairments were recruited at a location which will be identified if the article is published. Participants received financial compensation for their participation. All participants used their right dominant hand to complete the experiment and their handedness was measured by the Edinburgh Handedness Inventory (Oldfield, 1971). Each participant only performed a single experimental paradigm. All participants were naive to the purpose of the experiment and gave written informed consent in accordance with protocols approved by the Institutional Review Board at a location which will be identified if the article is published.

**Figure 1.**
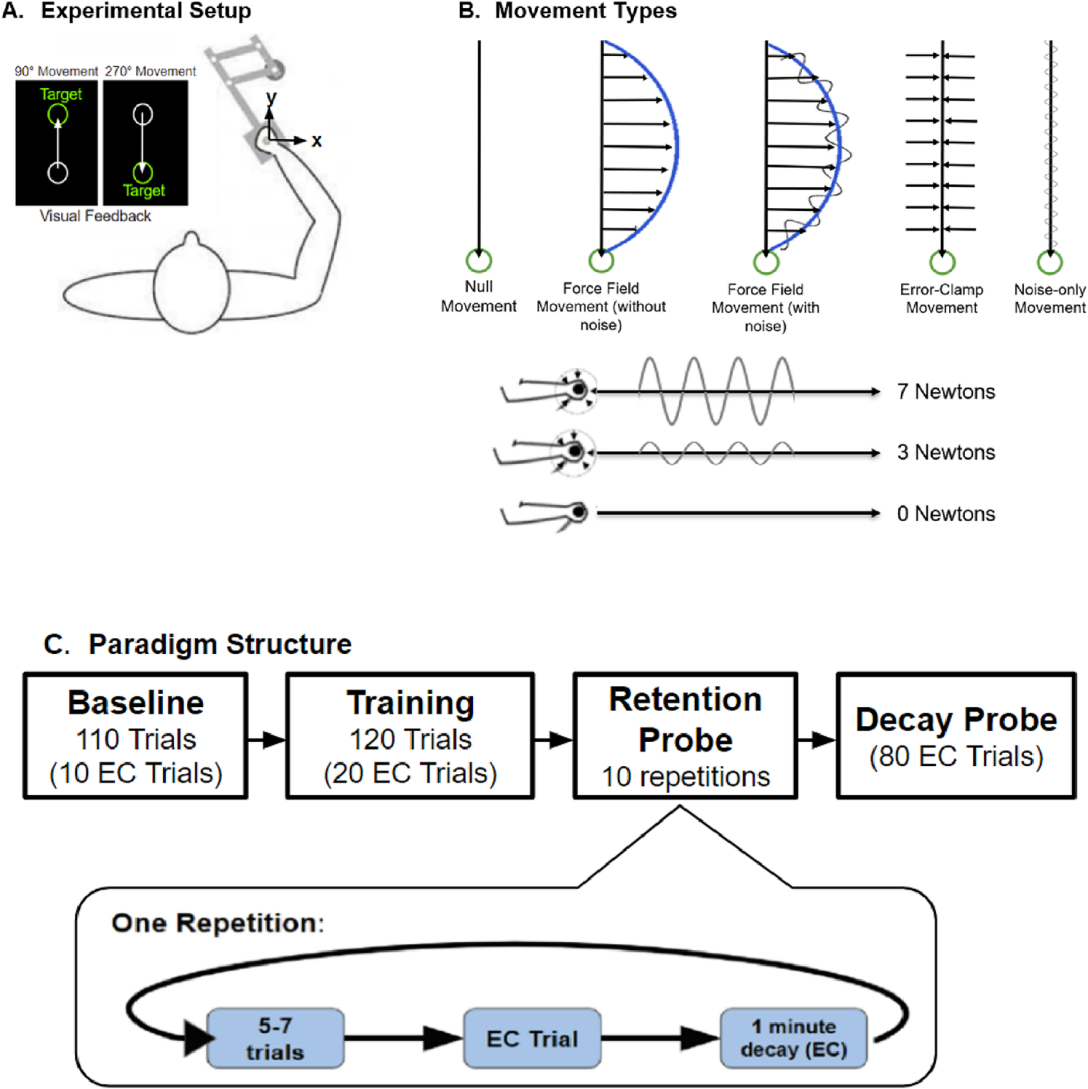
Experimental set-up and protocol. ***A***, Participants made straight arm reaching movements between two circular targets from midline ∼10 cm apart while holding the handle of a robotic manipulandum. Forward reaching motion was completed in the 90° direction while reaching back toward oneself was completed in the 270° direction. However, we only used arm movements completed in the 270° direction to compute adaptation coefficients and assess force profiles. The location of the hand was represented on the screen as a white filled circle. ***B***, There were four movement types: null, noise-only, force field (without noise), force field (with noise), and error-clamp movements. Each trial consisted of one movement. Null movements were completed as a free-range movement (no resistance from the robot) without any added noise or force field perturbation. Noise-only movements were free-range movements with added noise (according to condition). During force field movements, participants experienced a velocity-dependent, lateral perturbation (horizontal small black arrows). For the 270° reaching motion, the force-field perturbed the participant arm reach to the right. The control condition experienced this perturbation without any added noise [force-field movement (without noise)]. The experimental groups experienced this perturbation with the addition of either 3 N or 7 N of noise (applied at 10 Hz) supplied by the robot [force-field movement (with noise)]. This noise was generated by the sine function described in the Materials and Methods. During error-clamp trials (EC), lateral movement was constrained by clamps, constraining lateral error to <0.6 mm. ***C***, Paradigm structure. Participants started off with a block consisting of null movements with occasional error-clamp trials to establish baseline performance. During the training blocks, participants experienced the force-field perturbation (with added noise in the 3N  and 7 N noise groups). During the retention probe block, on some of the trials, participants were asked to hold the cursor at the start target for ∼1 min before proceeding with the reaching movement. During the decay probe block, participants were given a series of 80 error-clamp trials to assess the decay of adaptation.

Motor adaptation was investigated through an arm reaching paradigm (Shadmehr and Mussa-Ivaldi, 1994; Scheidt et al., 2000; Fig. 1). The paradigm required participants to move a robotic arm on a planar workspace that moved a screen cursor between presented targets. The robotic arm manipulandum (KINARM End-Point Lab, BKIN Technologies) sampled motor output, including position, force, velocity, and acceleration at 1,000 Hz. A horizontal screen display blocked participants from viewing their arm directly. A downward-facing LCD monitor, reflected by an upward-facing mirror, allowed viewing of trial start locations and targets, marked by small circles. Participants were seated in an adjustable chair so that they could comfortably view the mirrored display.

Participants were instructed to make straight arm reaching movements between two circular targets 0.5 cm in diameter spaced 10 cm apart on a screen while holding the handle of the robotic manipulandum (Fig. 1*A*). The two targets were located 20–30 cm away from the body on the sagittal axis. Arm movement via the robotic manipulandum was represented by a white filled circle, 3 mm in diameter on the screen. Horizontal and vertical arm movement (i.e., movement within the 2D plane that results in *x* and *y* movement directions of the cursor) was measured, and a successful movement to the target was achieved when the peak movement velocity in the *y* direction (toward the target) was within a range of 0.2–0.55 m/s. Additionally, participants were given both visual and auditory feedback on successful reaches. When peak movement velocity met the specific criteria, the target would turn green and make a brief auditory tone. When the reach was too slow, the target would turn yellow. If too fast, red. At the beginning of the task, participants were instructed to obtain “as many green feedback trials as possible.”

Within the task, there were four types of trials: null, force-field perturbation (FF), error-clamp, and noise-only (Fig. 1*B*). During null trials, participants made straight arm reaching movements between the targets without any perturbations or restrictions to movement (no noise and no force- field). During FF trials, reaching arm movements were systematically perturbed during the training period. On these trials, the robot perturbed the hand motion with forces that were proportional to the velocity of movement and perpendicular to the direction of hand motion (Eq. 1). Within the FF trials, there were three groups of subjects, and each group experienced a different level of noise, (magnitudes of either 0, 3, or 7 N, at a frequency of 10 Hz) in addition to the main FF perturbation. For the level of noise, we wanted to simulate the pathological tremor experienced by those with essential tremor. A 10 Hz frequency is within the range for that experienced by individuals with essential tremor. However, we were limited by the KINARM's generation of noise magnitude (i.e., force). Using our best estimates, we wanted to capture the full range of noise magnitude that could be produced by the KINARM without causing discomfort to participants. The 7 N magnitude was the far end of this range while the 3 N magnitude was approximately between 0 and 7 N. The noise was generated by a sine function (Eq. 2) and applied along the *x*-axis—the axis that subjects would eventually experience the force-field perturbation.

$$\left[\begin{array}{c} F_{x}\\ \;F_{y}\; \end{array}\right]=c_{k}\left[\begin{array}{c} 0\;-k\;\\ k\;\;0\; \end{array}\right]\left[\begin{array}{c} \dot{x}\;\\ \dot{y}\; \end{array}\right],\;k=15\;\;\mathrm{Ns}/m.$$

**Figure 2.**
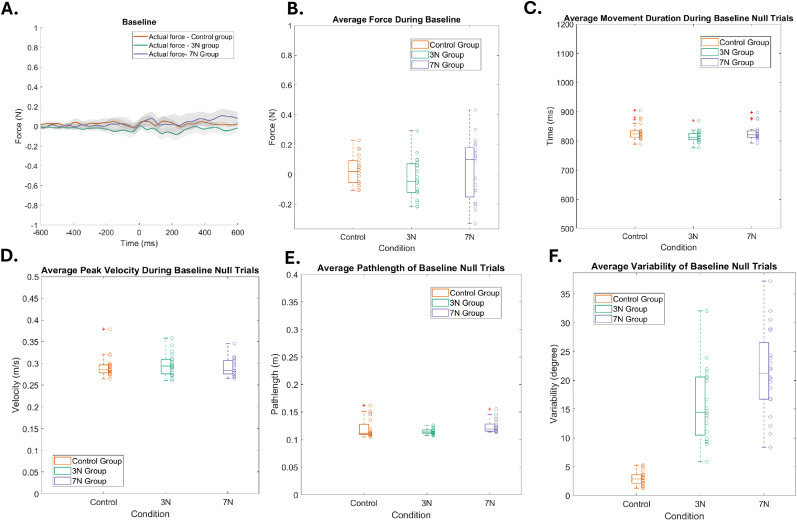
Comparison of baseline movements. ***A***, Force profiles comparison across conditions (orange, control group with no added noise; green, 3 N of added motor noise; purple, 7N of added motor noise). Force profile is the value, measured in magnitudes (N) on the *y*-axis, used to indicate how much lateral force was exerted during EC trials throughout a single reaching movement. Force profiles on individual trials were centered on peak velocity (peak velocity indicated as 0 ms). Box plots are shown for the average (***B***) force (***C***) movement duration, (***D***) peak velocity, (***E***) path length, and (***F***) variability of baseline null trials. Each unfilled circle represents the results for the average of the windowed trials. Force, movement duration, peak velocity, and path length were not significantly different across the three conditions. However, movement variability was significantly different at baseline.

Error-clamp (EC) trials assessed the feedforward adaptive changes in motor output. EC trials involved restricting lateral movement, forcing participants to move the cursor in a straight line toward the target so that lateral errors were kept to a minimum. In this case, the robot motors constrained movements in a straight line toward the reach target by counteracting any motion perpendicular to the target direction. This was achieved by applying a stiff one-dimensional spring (6 kN/m) and a damper (150 Ns/m) in the axis perpendicular to the reach direction. In these trials, perpendicular displacement from a straight line to the reach target was held to <0.6 mm and averaged ∼0.2 mm in magnitude. Importantly, the force field (and added noise) was not applied on these error-clamp trials. Thus, there would not be any rapid feedback correction on these error-clamp trials. The error-clamp trials provide the force profiles shown in Figures 2 and 4 (the force the robot must apply in order to ensure the subject moved in a straight line).

During noise-only trials, participants made straight arm reaching movements between the targets while the robot applied the amount of noise consistent with their condition (magnitudes of either 0, 3, or 7 N, at a frequency of 10 Hz).

Noise=m⋅sin(2⋅π⋅f⋅t),f=10Hz,m=0,3or7N.

### Experimental paradigm

The task began with a familiarization period, where participants of all groups had null (no added noise or FF) movement trials. One group (0 N noise) served as the control group. Two groups experienced noise with an amplitude of 3 or 7 N (except for during the familiarization period). This was followed by baseline trials. For the control group, the baseline block contained 100 null trials randomly dispersed with 10 EC trials (110 trials total). For the two noise groups, the baseline consisted of 50 noise-only trials and 60 null trials (no noise) randomly dispersed with 10 EC trials (120 trials total). Immediately following baseline, participants were exposed to a training block. As described above, we divided participants into three groups based on the amplitude of the noise experienced during the training period. Each participant only experienced one type of training and the training period consisted of 100 FF trials interspersed randomly with 20 EC trials (120 trials total). For every 5 FF trials, there was 1 EC trial randomly dispersed. In addition to the FF perturbation, the robot applied the magnitude of noise that corresponded to the condition of each participant.

The retention block (Fig. 1*C*) immediately followed the training period. During this block participants experienced 5–7 trials of the FF perturbation (with the added noise based on the subject group) followed by an EC trial. This EC trial was followed by a delay period of 1 min during which participants were asked to hold the cursor at the start location. Based on prior work, we hypothesized that during this delay, the fast process would rapidly decay so that the subsequent error-clamped movement on the trial following the delay would reflect the amount of slow learning process retained over the period without movement. This sequence (5–7 FF trials, 1 EC trial, a 1 min delay, and 1 EC trial) was repeated 10 times within the retention block. Finally, following the retention block there was a decay block, which consisted of 80 consecutive EC trials. This allowed a measure of the decay of adaptation toward baseline.

### Quantification of adaptation

To quantify adaptation throughout the task, we use the lateral force profiles measured throughout the EC trials. For our analyses, we only calculated adaptation and the applied force profiles for reaches in the direction toward the body (270°). Since we only perturbed arm reaching movement during the 270° movements, we wanted to analyze only that direction for changes in participant force profile. For each FF trial, there is an ideal temporal pattern of compensatory force to fully compensate for the lateral perturbation. This ideal compensatory force is directly proportional to the velocity profile of movement on that trial, so the ideal force profile for each FF trial is unique. Force profiles are the temporal pattern of applied force (N) which is measured continuously throughout a single reaching movement. The force profiles on individual trials were centered on the peak velocity with a temporal window of 1,200 ms (±600 ms, where 0 ms is the moment when the movement reaches peak velocity). This provides an alignment of all movements in the same temporal window. Force profiles were aligned to the peak velocity because the force-field perturbation applied by the robot is based on the movement velocity. Thus, plotting the ideal force pattern (based on the actual movement velocity on that trial) with the actual applied force on that trial provides a straightforward comparison of the two profiles. We calculated the participants’ ability to adapt to the perturbations by linearly regressing the baseline-subtracted actual force profile applied by participants to the ideal compensatory force profile for each EC trial. The baseline subtractions are subtracting the average force profile, determined over the same 1,200 ms window, obtained during the EC trials during the baseline period from the force profile on the EC trials experienced during training. Determining the regression slope involved utilizing a least squares estimate to minimize the model fit error, and the slope was used as the measure to quantify adaptation (i.e., the adaptation coefficient, AC). It is this slope of the linear regression between the baseline-subtracted actual force and the ideal force (based on the movement velocity on that trial) that is the scalar AC—a measure of how well the applied force matches the ideal force based on the movement velocity. If the actual compensatory force was equal to the ideal compensatory force, then the AC = 1. If they are exact opposites, AC = −1. If they are unrelated to one another, AC = 0.

### Baseline analyses

To better understand how the groups performed before exposure to the perturbation condition, we quantified the variability of null movements during the baseline period. In this case variability refers to the angular deviation from the straight line path at the point of maximum lateral movement deviation.

### Computational modeling

To determine the effect of the applied noise on specific learning mechanisms, we used the multi-rate gain-independent two-state model from Smith et al. (2006). The equations for adaptation to error (perturbation) are shown below.

*Two-State Model* (Smith et al., 2006):

$$x_{f}[n+1]\;=\;A_{f}\;\cdot x_{f}[n]\;+\;B_{f}\;\cdot \;e[n]\;,$$

$$x_{s}[n+1]=\;A_{s}\;\cdot x_{s}[n]\;+\;B_{s}\;\cdot \;e[n],$$

$$B_{f}\;>>\;B_{s}\;,\;A_{s}>>A_{f}\;,\;x[n]\;=\;x_{f}[n]\;+\;x_{s}[n],$$

$x_{f}[n]$, $x_{s}[n]$: Net motor output on trial n

$A_{f}$, $A_{s}\;$: Retention factors

$B_{f}$, $B_{s}$: Learning rates

e[n]: Error on trial n

*FF Trials*:

e[n]=f[n]−x[n],

*EC Trials*:

f[n]=x[n]→e[n]=0,

e(n): error on trial n

f(n): strength of FF disturbance on trial n

x(n): state of learned motor output on trial n

The two-state model postulates that adaptation is supported by two, distinct processes that operate in parallel (Eqs. 3, 4). The total compensation to error (x[n]) is the sum of the fast $\left(x_{f}[n]\right)$ and slow $\left(x_{s}[n]\right)$ learning processes. The fast and slow learning processes differ in their respective learning $\left(B_{f},B_{s}\right)$ and retention $\left(A_{f},A_{s}\right)$ rates. Here, learning rates are quantified by the previously mentioned adaptation coefficients, which are averaged across participants for each trial. The fast learning process has a faster learning rate than the slow learning process $\left(B_{f}>>B_{s}\right)$ but the slow learning process has a much greater retention factor $\left(A_{s}>>A_{f}\right)$. The net motor output on the next trial (*x*[*n* + 1], *A*) is dependent on the amount of learning retained (retention factor) scaled by the amount of the fast or slow process (the internal state). The amount of the fast or slow process has been intrinsically normalized based on the current motor output in addition to the current learning rate (*B*) scaled by the amount of error on the current trial (*e*[*n*]). The net motor output on a specific trial (*x_f_*[*n*], *x_s_*[*n*]) refers to the AC for each learning process (fast or slow). As the ACs themselves are scalar (−1 to 1), the net motor output reflects this learning in a scalar fashion. The potential amount of learning based on the presence or absence of error on the current trial, for the purposes of this study, was normalized as either f[n]=0 (no perturbation) or f[n]=1 (FF is present). In the FF trials, the strength of the perturbation (*f*[*n*]) minus the learned force to counteract that perturbation (*x*[*n*]) during a single trial will provide the amount of error (*e*[*n*]) that remains. Note that to determine the adaptation coefficient, we change the sign of the ideal force profile to have a direct comparison to the actual force profile. That is, the actual force should be opposite the FF, but we ensure that both are in the same direction (flip the sign) to determine how well they match. In this way, a positive coefficient represents a successful cancellation of the perturbation. Thus, in this case the error is *f*(*n*) − *x*(*n*). Additionally, *x_f_*[*n*] and *x_s_*[*n*] represent the respective amount of the adaptation to the force-field perturbation attributed to either process. When *f*[*n*] = 0 during EC trials, there is no potential for learning because the error is clamped to zero.

Model parameters were derived within groups (i.e., controls, 3 N, 7 N) by minimizing the model fit's error relative to the collected data with the *fmincon* function in MATLAB; Eq. 7). Because our optimization function is biased toward the asymptote, we wanted our initial parameters to more closely reflect the initial rise in the adaptation coefficient (*A_f_* = 0.775; *B_f_* = 0.06; *A_s_* = 0.992; *B_s_* = 0.02). To accurately constrain the best-fit parameter estimates of the data, we ran a bootstrapping algorithm that sampled participant data with replacement and added a regularization term to the error function to prevent biasing effects from repeated outliers in the data (Eq. 8). This procedure was replicated 2,000 times to reliably estimate a distribution of bootstrapped parameters. Each replicate was made by sampling data from 20 randomly generated choices generated from the 20 participants in the study. We made 2,000 different bootstrap estimates and fit the model to each estimate. We used the 2.5 and 97.5 percentile values of each parameter distribution's fitted Gaussian as the 95% confidence interval limits.

Estimating plausible ranges of estimates for each of the four model parameters ( $A_{f}$, $B_{f}$, $A_{s}$, $B_{s}$) happened in two steps. In Step 1, we computed the original parameter estimates based on the least-squares method. We first estimated reasonable values for each of the four model parameters by minimizing the model prediction error relative to the original data *y* (i.e., best model fit)*.* Specifically, we ran the *fmincon()* function in MATLAB to extract the parameter estimates that would minimize the error function as described in Equation 7.

$$\theta_{\mathrm{LMSE}}=\mathrm{argmin}\left\{\frac{\sum_{n=1}^{N}\;{\left(y^{\left(n\right)}-{\hat{y}}^{\left(n\right)}\right)}^{2}}{N}\;\right\},$$

where *N* refers to the total number of trials, $y^{\left(n\right)}$ refers to the original observation on trial *n*, and ${\hat{y}}^{\left(n\right)}$ refers to the model prediction on trial *n*.

In Step 2, we estimated plausible confidence intervals around the original parameters $\theta_{\mathrm{LMSE}}$. We implemented a bootstrapping strategy to estimate a reasonable confidence interval around each of the original LMSE parameters within $\theta_{\mathrm{LMSE}}$. This strategy consisted in sampling data with replacement and estimating new $\hat{\theta }$ vectors for each bootstrap. Bootstraps were made by concatenating 20 randomly generated choices corresponding to data produced by the 20 participants in the study. We extracted 2,000 different bootstrapped parameter estimates by fitting the model to each random data concatenation. We added a regularization term to Equation 7 to penalize extreme weights due to the biasing effect of occasional repeated outliers in the sampled data. We implemented the *fmincon()* function in MATLAB to estimate each of the 2,000 bootstrapped $\hat{\theta }$ based on Equation 8.

$$\hat{\theta }=\mathrm{argmin}\left\{\frac{\sum_{n=1}^{N}\;{\left(y^{\left(n\right)}-{\hat{y}}^{\left(n\right)}\right)}^{2}}{N}+\;\lambda \overset{4}{\underset{s=1}{\sum }}\;{\left[\frac{{\hat{\theta }}_{s}-\theta_{\mathrm{LMSEs}}}{\sqrt{\theta_{\mathrm{LMSEs}}}}\right]}^{2}\right\},$$

where *N* refers to the total number of trials, $y^{\left(n\right)}$ refers to the original observation on trial *n*, ${\hat{y}}^{\left(n\right)}$ refers to the model prediction on trial *n*, $\theta_{\mathrm{LMSE}}$ refers to the original model parameters from Step 1, and λ refers to a regularization constant which was set to λ=0.05. This choice of value for the hyperparameter λ was found to be a reasonably low option that optimizes the trade-off between data overfitting (yielding $\hat{\theta }$ outliers) and the overly constrained normalization of the $\hat{\theta }$ distribution. Note, here, that for each parameter estimate *s* the choice of $\sqrt{\theta_{\mathrm{LMSEs}}}$ as the denominator in the regularization term ensured that all four ${\hat{\theta }}_{s}$, lying between 0 and 1, would equally be constrained by the regularization factor λ regardless of their relative magnitude.

For the bootstrapping algorithm, we used the following bounds in the initialization of the *fmincon()* function across all conditions:

$$A_{f}:0.25\text{–}0.75,$$

$$B_{f}:0.0\text{–}0.2,$$

$$A_{s}:0.93\text{–}1.0,$$

$$B_{s}:0.0\text{–}0.2,$$

This method was replicated 2,000 times in each bootstrap to estimate four $\hat{\theta }$ distributions. A normal Gaussian was fitted to each of these distributions to estimate each parameter's mean and 95% confidence interval. Specifically, we used the 2.5 and 97.5 percentile values of each parameter as the 95% confidence interval lower and upper bounds.

### Code accessibility

The code/software described in the paper is freely available online at osf.io/cktnw. The code is available as Extended Data.

### Statistical analysis

Statistical analyses were performed in MATLAB (version R2023a) on trials that met the speed, duration, and position criteria listed above. Based on these criteria, 5% of trials were discarded for the control group, 1.8% of trials were discarded for the 3 N group, and 5.45% of trials were discarded for the 7 N group. In our statistical analyses, we used both fixed (i.e., condition) and random (i.e., subjects) effects. To compare the learning rate for each noise condition, we utilized a logistic growth model (Eq. 9, listed in results) using trial number and average adaptation coefficient across participants for each trial number. We then calculated the *R*-squared value for goodness of fit for our data. We hypothesized that the control group would have a greater maximum adaptation and would reach this asymptote at a faster rate than the 3 N and 7 N groups. To compare force profiles across the force windows (150 ms before peak movement, peak movement, and 150 ms after peak movement) and period of training (early, middle, and late training) for each noise condition, in addition to comparing average adaptation coefficient for each period of training across groups, we utilized a two-way and three-way analysis of variance (ANOVA). We predicted that the adaptation would be greater for the control group than the 3 N and 7 N conditions during middle and late training due to the 3 N and 7 N groups having impaired learning during early training. For the retention trials, we compared the average adaptation coefficient at the end of training and the average adaptation coefficient during the retention probe across groups with a two-way ANOVA. Due to our overall hypothesis of an unimpaired slow learning process, we predicted that there would not be a significant difference between the end of training and retention probe performance across groups. If significant results were found, we further investigated significance between groups. Additionally, to confirm statistical equivalence across all groups for retention probe performance, we conducted Mann–Whitney *U* tests. To better compare the differences in the average amount of decay across motor noise conditions, we performed a two-phase exponential fit (Eq. 10, listed in results), using trial number and average adaptation coefficient across participants for each trial number, and compared the exponential decay constants. To again address our overarching hypothesis that long-term retention, driven by the slow learning process, was not impaired by the added noise, we predicted that the rate of decay would show no statistical difference across conditions. We conducted an Akaike information criterion (AIC) test to determine the optimal model, specifically evaluating the appropriateness of one-phase and two-phase exponential decay models. We computed the log-likelihood from fits to the one-phase and two-phase exponential decay models by fitting the data under a *t*-distribution and basing the AIC on the sum of the squared errors (Akaike, 1998). The results of the goodness-of-fit assessment revealed that the two-phase exponential decay model provided the most favorable fit for our dataset. For all ANOVAs, we corrected for multiple comparisons using Bonferroni’s post hoc test. For significant interactions, we conducted post hoc Tukey-HSD tests. Effect size (eta squared) was calculated using the hhentschke/measures-of-effect-size-toolbox in MATLAB (Hentschke, 2023). For all tests, the significance level was 0.05 and data are presented by means ± SE.

## Results

In this study we investigated the specific mechanisms by which increasing externally applied motor noise magnitude affected motor adaptation. We utilized a force-field adaptation task (Shadmehr and Mussa-Ivaldi, 1994) in which three groups of participants were exposed to varying levels of noise magnitude (0, 3, or 7 N, at a frequency of 10 Hz) while being simultaneously perturbed by the velocity-dependent force-field perturbation. Each group consisted of 20 individuals. Each individual only experienced one noise level within the experiment. We examined the effects of the externally applied noise on the time course of adaptation, as well as the retention and decay of the learning.

### The effect of motor noise on unperturbed and perturbed movements

To determine if the three groups differed during baseline movements (prior to any training), we examined force profiles during EC trials in addition to the movement duration, peak velocity, and path length of the null trials. Average force profiles were not significantly different across the three conditions (Fig. 2*A*,*B*; one-way ANOVA; *F*_(2,59)_ = 0.96; *p* = 0.39; *η*^2^ = 0.03). However, movement duration (Fig. 2*C*; one-way ANOVA; *F*_(2,59)_ = 2.06; *p* = 0.14; *η*^2^ = 0.68), peak velocity (Fig. 2*D*; one-way ANOVA; *F*_(2,59)_ = 0.35; *p* = 0.7; *η*^2^ = 0.01), and path length (Fig. 2*E*; one-way ANOVA; *F*_(2,59)_ = 2.37; *p* = 0.1; *η*^2^ = 0.08) of baseline null trials were not significantly different across conditions. Variability of baseline null trials was significantly different between conditions (Fig. 2*F*; one-way ANOVA *F*_(2,59)_ = 39.2; *p* = 1.99 × 10^−11^; *η*^2^ = 0.58), with the 7 N group having greater pretraining variability followed by the 3 N and 0 N groups (see Materials and Methods). Thus, the added noise affected certain aspects of movement kinematics on the baseline trials. Next, we examined these effects on movement adaptation in response to a velocity-dependent force-field perturbation.

### Added motor noise affects the initial rate of adaptation

**Figure 3.**
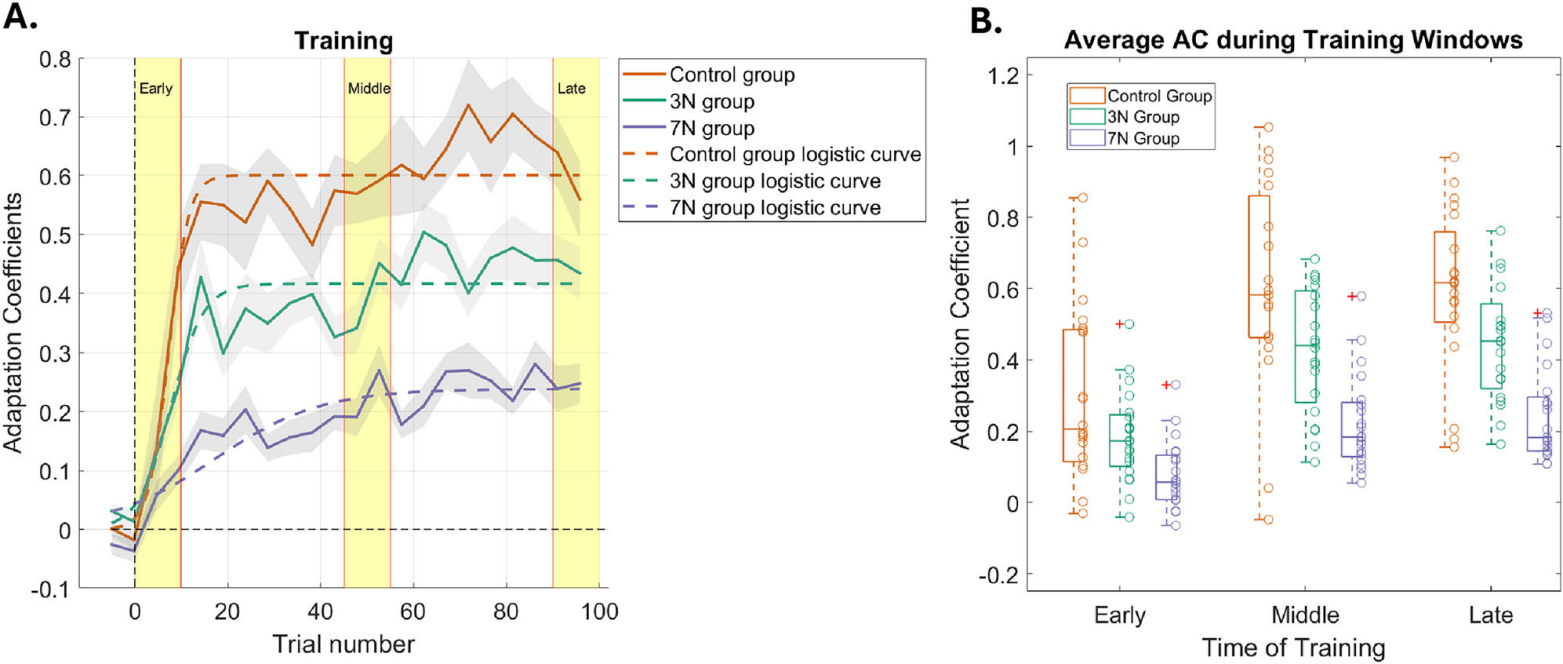
Learning curves during training. ***A***, Adaptation, quantified by the adaptation coefficient, is plotted as a function of trial number for each condition. These learning curves show that as noise increases (control, orange trace; 3 N, green trace; 7 N, blue trace), the ability to adapt becomes impaired. Logistic growth curve fits (dashed traces) for each condition are shown: *R*^2^_control _= 0.92, *R*^2^_3N _= 0.85, *R*^2^_7N _= 0.78. Gray shading represents SEM. Yellow regions mark the early, middle, and late periods of training. ***B***, Box plots of the average adaptation coefficient for each condition during early, middle, and late periods of training for each group. Each unfilled circle represents the results for one subject.

Figure 3*A* plots the adaptation coefficient (see Materials and Methods) as a function of training trials for the three subject groups. To quantify the learning rate of each motor noise condition, we utilized a logistic growth model, shown below:

$$A_{n}=\;\frac{K}{1\;+\;\left(\frac{K-A_{0}}{A_{0}}\right)e^{-rn}},$$

$A_{n}$: adaptation coefficient on trial *n*

K: carrying capacity (maximum adaptation coefficient subjects could reach)

$A_{0}$: initial adaptation coefficient

r: growth rate for learning curve

The control group (0 N of applied motor noise) achieved the greatest maximum adaptation (*K*_control _= 0.60; 95% CI [0.57 0.63]) and did so at the fastest rate (*r*_control _= 0.37; 95% CI [0.15 0.59]; *R*^2 ^= 0.92; Fig. 3*A*). The 3 N group achieved a maximum adaptation of *K*_3N _= 0.42, 95% CI [0.39 0.45] with a learning rate that was slower than the control group (*r*_3N _= 0.26; 95% CI [0.08 0.44]; *R*^2^ = 0.85). The 7 N group achieved the lowest maximum adaptation (*K*_7N _= 0.24; 95% CI [0.20 0.28]) with the slowest earning rate (*r*_7N _= 0.07; 95% CI [0.02 0.12]; *R*^2^ = 0.78). These results suggest that as motor noise magnitude increased, the ability of each group to adapt decreased (*K*_control _> *K*_3N_ > *K*_7N_). Additionally, increasing motor noise magnitude also affected the rate at which peak adaptation occurred, with higher magnitudes of motor noise associated with slower learning rates (*r*_control _> *r*_3N _> *r*_7N_).

To better understand how the average adaptation coefficients differed at specific time periods of training, we examined the average adaptation coefficient from the first 10% of training trials (early trials), the middle 10% (middle trials), and last 10% (late trials; Fig. 3*B*). A two-way ANOVA demonstrated that there was a significant difference in adaptation between motor noise groups (*F*_(2,178)_ = 25.89; *p* = 1.53 × 10^−10^; *η*^2^ = 0.2), and within each motor noise group, adaptation was significantly different between three training periods (two-way ANOVA; *F*_(2,178)_ = 26.07; *p* = 1.33 × 10^−10^; *η*^2^ = 0.2). No significant interaction between the two factors was found (*F*_(4,178)_ = 1.53; *p* = 0.2; *η*^2^ = 0.02). The decrease in learning rate occurred early in training; in the early training period, the control group [orange trace; adaptation coefficient (AC) of 0.24 ± 0.10] has a steep rise whereas the 3 N (0.19 ± 0.03) and 7 N (0.08 ± 0.02) groups had not adapted as well (green and purple traces, respectively). The slowed and impaired ability to adapt in early trials due to increasing motor noise magnitude is subsequently followed by lower performance overall in the middle and later trials (middle training period: control, 0.61 ± 0.07; 3 N, 0.44 ± 0.04; and 7 N, 0.23 ± 0.03; late training period: control, 0.6 ± 0.05; 3 N, 0.44 ± 0.04; and 7 N, 0.24 ± 0.03).

### Added motor noise affects the temporal force profiles

**Figure 4.**
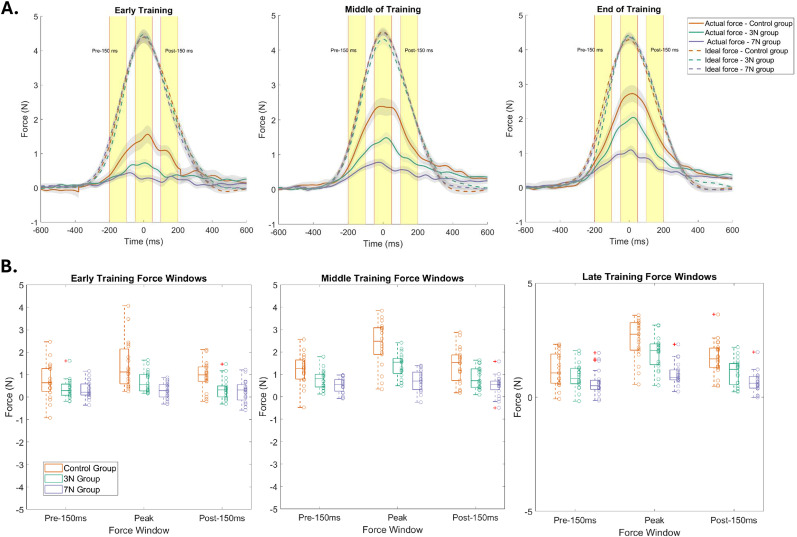
Temporal force profiles during training. ***A***, Average force profiles for each group during early, middle, and late periods of training for the three conditions (orange, control group with no added noise; green, 3 N of added motor noise; purple, 7 N of added motor noise). Force profile is the value, measured in magnitudes (N) on the *y*-axis, of force used to counteract the FF perturbation throughout the reaching movement. Ideal force profile represents how much force would be needed to exactly counteract the FF perturbation to continue making a straight arm reaching movement. The actual force profile indicates how much force, on average, participants used to counteract the FF perturbation. Force profiles were split into three windows: one 100 ms window centered around 150 ms prior to peak movement velocity, one 100 ms window centered around the peak (time = 0) movement velocity, and one 100 ms window centered around 150 ms after peak movement velocity. These three time periods are represented by the three yellow regions. ***B***, Box plots show differences in the average force profile for each condition during the pre-150 ms force window, peak force window, and post-150 ms force window within early, middle, and late periods of training.

We analyzed the respective force profiles within a 100 ms window to identify differences between the temporal structures of adaptation (Joiner et al., 2017). This also provided information on the effect of increasing noise magnitude on adaptation reflected in the force profiles across conditions (Fig. 4). We continued to separate the training data into early, middle, and late trials, as described previously.

To best represent the differences between the temporal force profiles, we also separated the data to compare the applied force within a 100 ms window centered on peak velocity, 150 ms before peak velocity, and 150 ms after peak velocity for each training period (Fig. 4*A*). There was a significant effect of training period (three-way repeated-measures ANOVA; *F*_(2,530)_ = 59.60; *p* = 5.49 × 10^−24^), temporal force window (three-way ANOVA; *F*_(2,530)_ = 31.98; *p* = 8.21 × 10^−14^), and noise condition (three-way ANOVA; *F*_(2,530)_ = 58.70; *p* = 1.14 × 10^−23^) on average force profile. These results verify that the difference in performance, as indicated by force profile, between the three conditions is dependent on specific periods of training, namely, middle and late training periods (explored in post hoc tests). Additionally, the control group had significantly greater peak force (2.32 ± 0.16) across training windows than the 3 N (1.43 ± 0.1) and 7 N (0.72 ± 0.08) groups, which suggests that increasing the magnitude of artificially applied noise impairs one's ability to accurately adapt. All three factors had significant interaction effects, which we further explored by looking at each training period (Table 1) and conducting post hoc tests. Post hoc Tukey-HSD tests revealed that there was greater force exerted in the 0 N middle training period (0.84 ± 0.76) than in the 3 N middle training period (0.54 ± 0.43; 95% CI [0.29 1.02]; *p* = 8.4 × 10^−7^) and in the 7 N middle training period (0.32 ± 0.22; 95% CI [0.72 1.46]; *p* = 9.76 × 10^−20^). Moreover, there was greater force exerted in the 3 N middle training period than the 7 N middle training period (95% CI [0.12 0.98]; *p* = 0.002). Peak force was overall significantly different between the 0 N and 3 N conditions (95% CI [0.37 1.23]; *p* = 3.28 × 10^−7^), the 0 N and 7 N conditions (95% CI [0.92 1.79]; *p* = 3.31 × 10^−22^), and the 3 N and 7 N conditions (95% CI [0.12 0.98]; *p* = 0.002). Further testing revealed that the peak force was specifically greater during the middle training period than the early training period (95% CI [−1.3 −0.89]; *p* = 8.86 × 10^−9^) but there was no significant difference in peak force between the middle and late training period.

**Table 1.** Mean and standard error for actual force throughout training

		Early	Middle	Late
Control	Pre-150 ms	0.42 ± 0.34	1.21 ± 0.16	1.21 ± 0.17
	Peak	1.11 ± 0.4	2.36 ± 0.23	2.65 ± 0.23
	Post-150 ms	0.7 ± 0.28	1.42 ± 0.18	1.73 ± 0.17
3 N	Pre-150 ms	0.38 ± 0.09	0.78 ± 0.09	0.91 ± 0.12
	Peak	0.69 ± 0.10	1.40 ± 0.12	1.94 ± 0.16
	Post-150 ms	0.35 ± 0.10	0.85 ± 0.11	1.10 ± 0.13
7 N	Pre-150 ms	0.33 ± 0.09	0.51 ± 0.08	0.65 ± 0.12
	Peak	0.29 ± 0.08	0.71 ± 0.10	1.02 ± 0.11
	Post-150 ms	0.26 ± 0.10	0.49 ± 0.10	0.66 ± 0.10

Mean ± standard error of the actual force at different periods in training (early, middle, and late) and at different force windows (at peak velocity, and 150 ms before and after peak velocity) within each noise condition.

During the early training period (Fig. 4, left column), there was a significant effect of noise group on average force profile (two-way ANOVA; *F*_(2,179)_ = 3.69; *p* = 0.03; *η*^2^ = 0.04), but the force window did not have a significant effect (two-way ANOVA; *F*_(2,179)_ = 0.94; *p* = 0.5; *η*^2^ = 0.01). There was no significant interaction effect between the two factors (two-way ANOVA; *F*_(4,179)_ = 0.89; *p* = 0.5; *η*^2^ = 0.02). Thus, regardless of temporal force window, as the magnitude of noise increased during the early training period, the average force profile decreased (control, 0.34 ± 0.21; 3 N, 0.29 ± 0.06; and 7 N, 0.17 ± 0.06).

During the middle training period (Fig. 4, middle column), there was a significant effect of temporal force window (two-way ANOVA; *F*_(2,170)_ = 19.56; *p* = 2.47 × 10^−8^; *η*^2^ = 0.13) and noise level (two-way ANOVA; *F*_(2,170)_ = 45.21; *p* = 2.5 × 10^−16^; *η*^2^ = 0.3) on the average force profile. Therefore, noise level affected specific temporal force windows. There was a significant interaction effect between the two factors (two-way ANOVA; *F*_(4,170)_ = 3.05; *p* = 0.02; *η*^2^ = 0.04), which we further explored by looking at each noise level (Table 1) and by conducting a post hoc test. Post hoc Tukey-HSD tests revealed that the 0 N condition had a greater amount of peak force (2.35 ± 1.02) than the 3 N condition (1.4 ± 0.54; 95% CI [0.34 1.57]; *p* = 4.64 × 10^−5^) and the 7 N condition (0.7 ± 0.46; 95% CI [1.03 2.27]; *p* = 2.15 × 10^−15^). Additionally, the 3 N condition had a significantly greater amount of peak force than the 7 N condition (95% CI [0.09 1.3]; *p* = 0.01). With this, we demonstrate that as the magnitude of noise increased during the middle training period, the average force profile decreased, and this variation was modulated throughout the force profile (Table 1).

During the late training period (Fig. 4, right column), there was also a significant effect of both temporal force window (two-way ANOVA; *F*_(2,179)_ = 32; *p* = 1.57 × 10^−12^; *η*^2^ = 0.2) and noise level (two-way ANOVA; *F*_(2,179)_ = 38.76; *p* = 1.31 × 10^−14^; *η*^2^ = 0.24) on the average force profile. Thus, noise level affected specific temporal force windows. There was a significant interaction effect between the two factors (two-way ANOVA; *F*_(4,179)_ = 3.33; *p* = 0.01; *η*^2^ = 0.04). Post hoc Tukey-HSD tests revealed that the 0 N condition had a greater peak force (2.65 ± 1.03) than the 3 N condition (1.94 ± 0.7; 95% CI [0.05 1.38]; *p* = 0.02) and the 7 N condition (1.02 ± 0.5; 95% CI [0.97 2.3]; *p* = 3.81 × 10^−13^). Moreover, the 3 N condition had a significantly greater peak force than the 7 N condition (95% CI [0.26 1.58]; *p* = 0.001). Similar to the middle of the training period, this suggests that as the magnitude of noise increased during the late training period, the average force profiles decreased and that this difference changed throughout the force profile (Table 1).

Overall, these results support the previous finding that increasing the magnitude of externally generated noise impairs adaptation to the force-field perturbation. Across all training windows, higher magnitudes of noise significantly reduced the force profiles, especially during peak movement velocity.

### Added motor noise affects the fast learning process underlying motor adaptation

We hypothesized that increasing the externally applied motor noise magnitude impaired adaptation by specifically affecting the fast learning process while the slow learning process remained largely unaffected. This can be attributed to the fast learning process, due to it having greater influence during early adaptation. Thus, the impairment of the initial steep increase in adaptation (Fig. 3) suggests that the fast learning process is impaired by the increase in motor noise magnitude.

**Figure 5.**
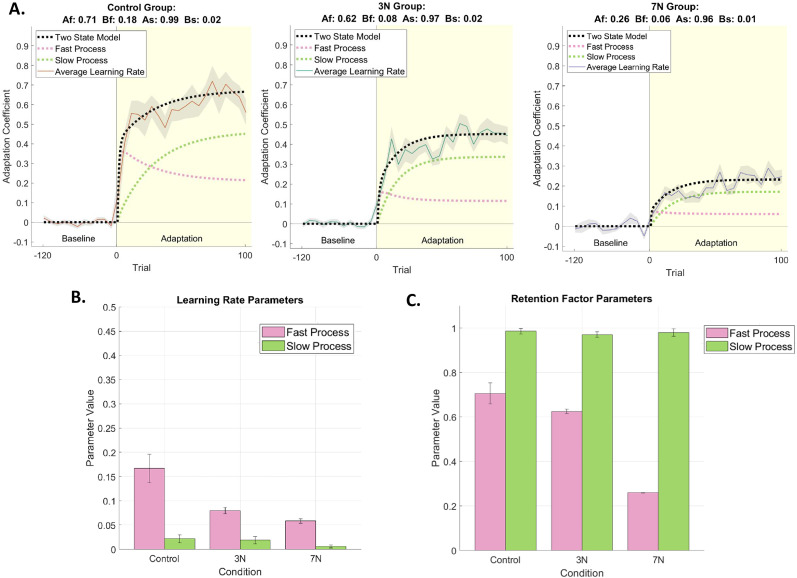
Two-state model results. ***A***, The overall adaptation (black trace), fast process (pink trace) and slow process (green trace) are shown for the three conditions (orange, control group with no added noise; green, 3 N of added motor noise; purple, 7 N of added motor noise). Colored traces represent the mean adaptation coefficient and gray shading represents SEM. Best-fit model coefficients are stated at the top of each graph. As noise magnitude increases, the learning rate for the fast process (*B_f_*) decreases while the learning rate for the slow process (*B_s_*) remains relatively unimpaired. ***B***, Learning rate parameters for each condition. As noise magnitude increases, the fast learning process rate decreases while the slow learning process rate remains relatively unimpaired. ***C***, Retention factor parameters for each condition. Retention for the fast learning process decreases as noise magnitude increases. However, retention in the slow learning process appears unaffected by the addition of motor noise. Error bars represent the 95% confidence intervals.

To test this hypothesis, we applied the two-state model to our data (Fig. 5*A*). In Figure 5, the overall adaptation rate for each group is represented by a solid black line (95% confidence intervals, control [0.66 0.71], 3 N [0.38 0.42], 7 N [0.18 0.23]). The slow process learning rate, indicated by the dotted green line in Figure 5*A*, is relatively unimpaired across conditions (parameter values for slow learning rate across groups, control *B_s_* 0.02, 3 N *B_s_* 0.02, 7 N *B_s_* 0.01; 95% confidence intervals, control *B_s_* [0.01 0.03], 3 N *B_s_* [0.01, 0.03], 7 N *B_s_* [0.003 0.01]; Fig. 5*B*). Additionally, the retention rates for the slow process (Fig. 5*C*) are unchanged (control *A_s_* = 0.99, 3 N *A_s_* = 0.97, 7 N *A_s_* = 0.96; 95% confidence intervals, control *A_s_* [0.97 1.0], 3 N *A_s_* [0.96 0.98], 7 N *A_s_* [0.96 1.0]. Conversely, the fast process learning rate, indicated by the dotted pink line in Figure 5*A*, is impaired by increasing motor noise magnitude (control *B_f_* 0.18, 3 N *B_f_* 0.08, 7 N *B_f_* 0.06; 95% confidence intervals, control *B_f_* [0.14 0.2], 3 N *B_f_* [0.07 0.09], 7 N *B_f_* [0.05 0.06]). These modeling results are summarized in Figure 5*B* and Table 2. Thus, application of the two-state model confirms that increasing the magnitude of externally applied motor noise impairs adaptation specifically by influencing the fast learning process (Fig. 5, pink bars) while the slow learning process remains relatively unaffected (Fig. 5, green bars).

**Table 2.** 95% Confidence intervals for two-state model parameters

	*A_f_* 95% CI	*A_s_* 95% CI	*B_f_* 95% CI	*B_s_* 95% CI
Control group	[0.66 0.75]	[0.97 1.0]	[0.14 0.20]	[0.01 0.03]
3 N group	[0.61 0.63]	[0.96 0.98]	[0.07 0.09]	[0.01 0.03]
7 N group	[0.26 0.26]	[0.96 0.99]	[0.05 0.06]	[0.003 0.01]

### Added motor noise does not affect short-term retention of motor adaptation

**Figure 6.**
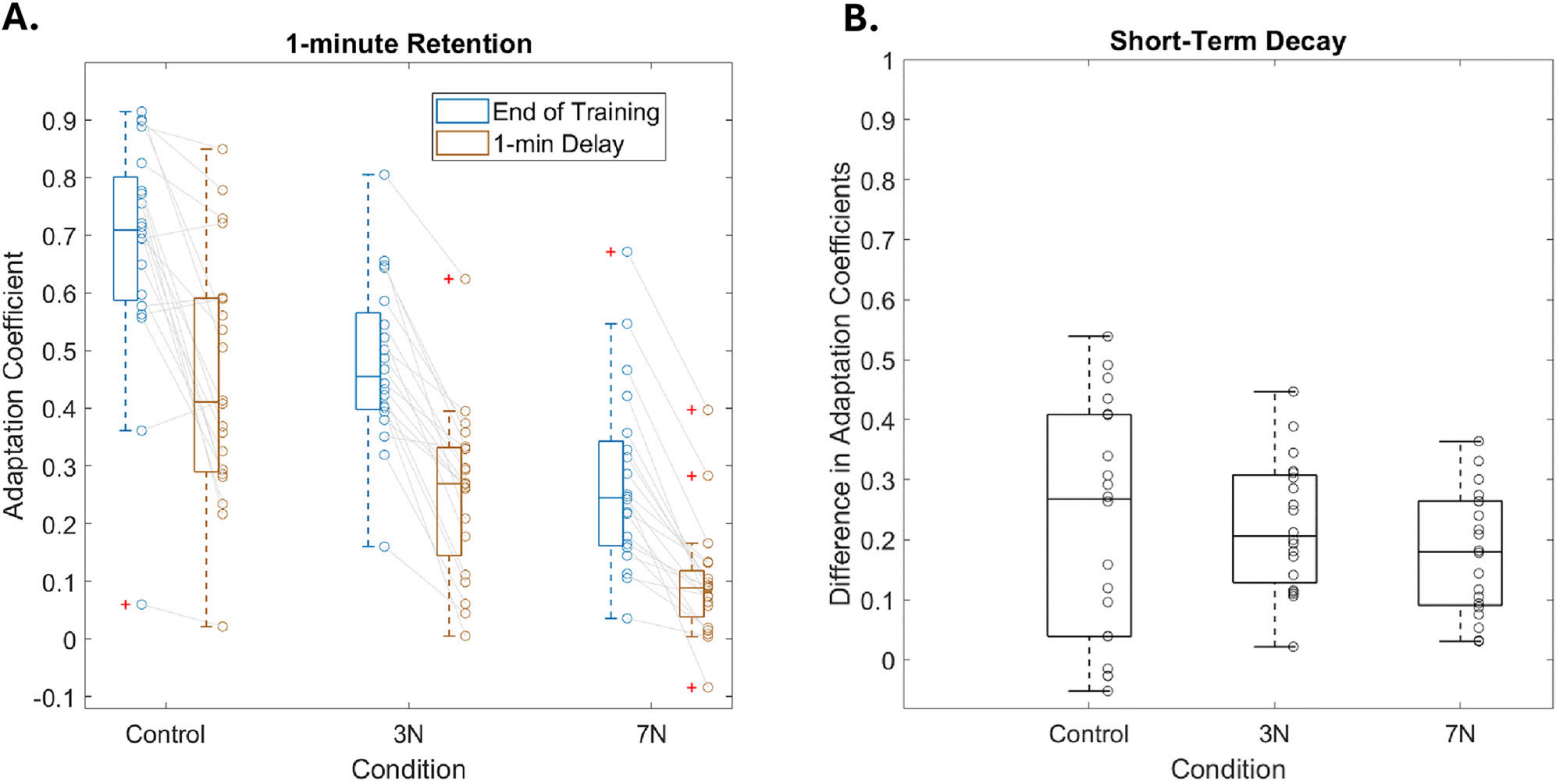
Retention of adaptation. During the retention probe, participants were initially re-exposed to the force-field movements, which was followed by error-clamp trials. ***A***, The average adaptation coefficients on these trials (blue box plots and circles) were compared with the average adaptation coefficients on the retention trials which followed a 1-minute delay period (red box plots and circles). Each unfilled circle represents the results for one subject. ***B***, Amount of short-term decay (the difference between the amount of adaptation at the end of training and after the 1 min delay period) for each condition.

We next examined the extent to which retention was affected by increasing motor noise magnitude, as measured by the 1 min delay probe during the retention block of the experiment (see Materials and Methods). One of the benefits of applying the two-state model to our results is the predictions we can make about behavior under different conditions. A prediction of a systematically impaired fast learning process (with the increase in noise) is that we should observe less of a decrease in overall learning over a 1 min hold period. This is due to the fast learning process decaying with time while the slow learning process maintains learning amounts well with the passage of time. Thus, by examining the difference in the learning amount before and immediately following the 1 min delay, we can indirectly assess changes in the fast learning process. During the 1 min delay, we expected that adaptation would rapidly decay by a set amount due to the time dependence of the fast process. Thus, during the error-clamp trial following the delay period, we could isolate the amount of adaptation that was retained due to the (relative) time-independence of the slow process. Figure 6*A* shows the adaptation coefficient before and after the 1 min delay for each subject group. There was a significant difference in the amount of adaptation between the end of the retraining period and the amount of adaptation after the 1 min delay period (two-way ANOVA; *F*_(1,119)_ = 119.23; *p* = 1.97 × 10^−19^; *η*^2^ = 0.44) that was significantly different across motor noise conditions (*F*_(2,119)_ = 12.1; *p* = 1.71 × 10^−5^; *η*^2^ = 0.09). These results suggest that there was a significant amount of decay between the end of the retraining period and the decay period and that this was true across noise levels. There was also a significant interaction effect between these two factors (*F*_(2,119)_ = 6.32; *p* = 0.003; *η*^2^ = 0.05). A post hoc Tukey-HSD test confirmed that there is no significant difference between the amount of adaptation during the 1 min delay between the control group and the 3 N group (*p* > 0.05). However, there is a significant difference between the control group and 7 N group (*p* = 0.03) as well as the 3 N group and 7 N group (*p* = 0.01). We do see that the mean difference over the 1 min hold period is in fact decreasing with noise level (0.45 ± 0.05, 0.26 ± 0.03, and 0.1 ± 0.02, for 0, 3, and 7 N respectively)—there is less of the fast learning process to decay with the increase in noise. Importantly, based on the model predictions, this difference should be minimal because the contributions of the fast learning process at asymptotic learning should be small compared with the slow learning process. That is, our intent was not to determine if there were significant differences between the subject groups, but rather to confirm that there is very little difference because the decay over the hold period is largely caused by the fast learning process.

Next, we determined if there was a significant difference in the amount of decay across noise conditions. We were interested in the amount of decrease in learning over the 1 min delay. Since the two-state model predicted that a decrease in the overall learning over a 1 min hold period should be attributed to the fast learning process, we plotted the difference in the adaptation coefficient before and immediately after the delay period. We wanted to determine if this decrease was roughly equivalent between groups despite there being significant differences in the level of adaptation before the delay. As reflected in Figure 6*B*, there was not a difference in the short-term retention of adaptation (difference before and after the 1 min delay; control, 0.23 ± 0.04; 3 N, 0.22 ± 0.02; 7 N, 0.18 ± 0.02). We found that the amount of reduction in the adaptation over the 1 min delay was not significantly different across conditions (one-way ANOVA; *F*_(2,59) _= 0.75; *p* = 0.5; *η*^2^ = 0.03). By examining the delta, we can postulate that the slow learning process is largely unaffected; even though the learning amount before the delay is different across the three conditions, the drop over the 1 min hold is comparable, suggesting learning is decaying to the level achieved by the slow learning process in each respective group. A Mann–Whitney *U* test showed that the control group (Mean ± SE: 0.23 ± 0.04) did not have significantly different decay from the 3 N group (0.22 ± 0.02), *z* = 0.07, *p* = 0.95, nor the 7 N group (0.18 ± 0.02), *z* = 0.83, *p* = 0.41. Additionally, the 3 N group and 7 N did not have significantly different decay (Mann–Whitney *U* test; *z* = 1.26; *p* = 0.21). The similarities in the reduction suggest that increasing motor noise magnitude does not significantly impair the short-term retention of adaptation.

### Added motor noise does not affect the decay of motor learning

**Figure 7.**
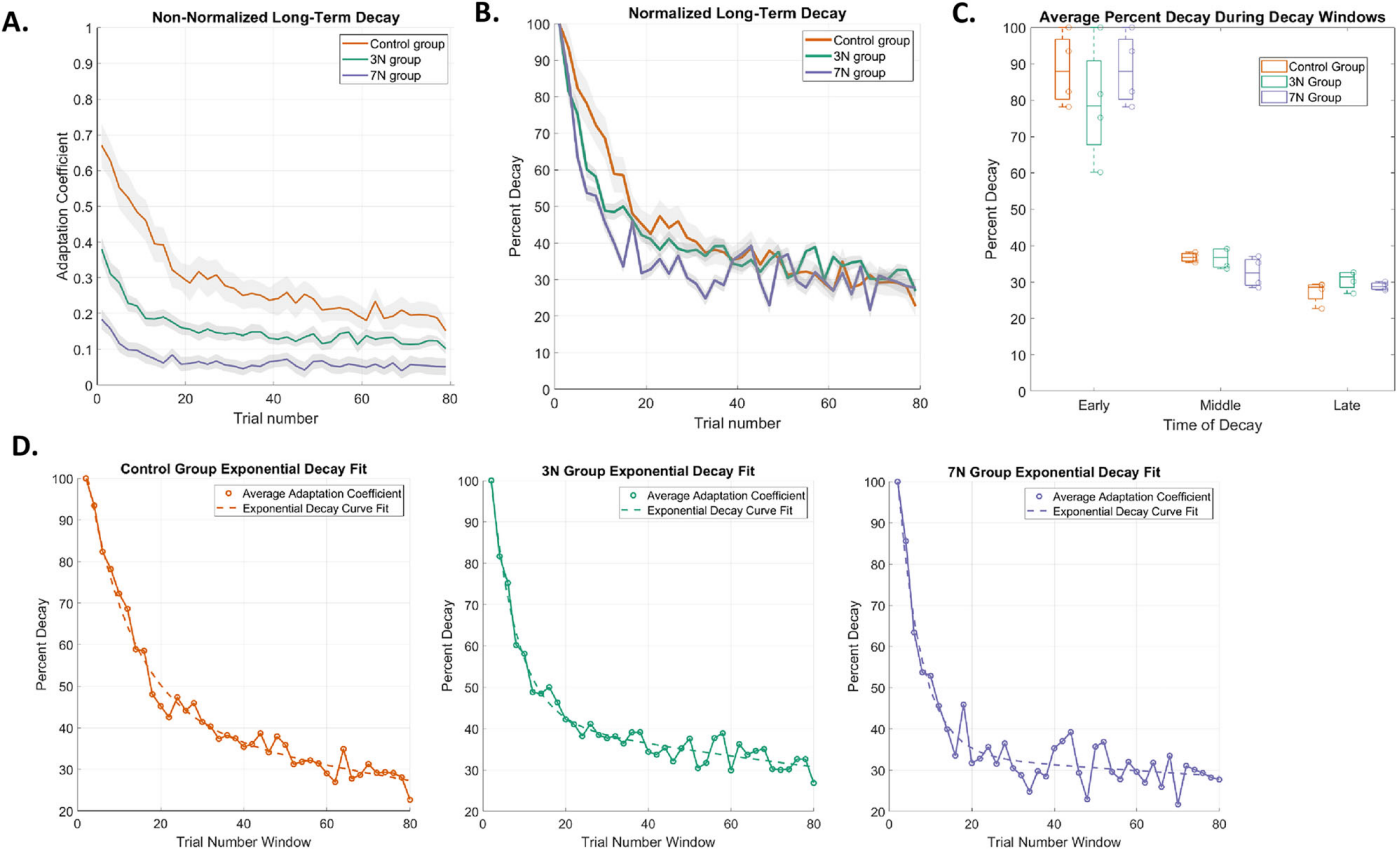
Decay of adaptation. ***A***, Non-normalized decay of adaptation, quantified by the raw adaptation coefficient, is plotted as a function of trial number for each condition (control, orange trace; 3 N, green trace; 7 N, blue trace) during the decay probe block. Colored traces represent the mean adaptation coefficient and gray shading represents SEM. ***B***, The normalized decay curves, quantified by the adaptation coefficient as a percentage of the value at the end of training, demonstrate that decay rates are similar across groups. The curves were normalized by dividing the average adaptation coefficient of each trial by the average adaptation coefficient for each subject during the first trial of the decay period. ***C***, Box plots of the average adaptation coefficient for each condition during early, middle, and late periods of decay for each group. Each unfilled circle represents the results for one subject. ***D***, Two-phase exponential decay fit, $Ae^{bx}+Ce^{dx}$, for each condition is shown: control, $70.91\times 10^{-0.18x}+44.08\times 10^{-0.01x}$ (*R*^2^_control _= 0.98); 3 N, $78.61\times 10^{-0.32x}+42.8\times 10^{-0.009x}$ (*R*^2^_3N _= 0.97); 7 N, $96.38\times 10^{-0.36x}+34.17\times 10^{-0.004x}$ (*R*^2^_7N _= 0.93). Adaptation coefficient is shown as a percentage of the value at the end of training.

In addition to retention, we also investigated the effect of motor noise on the decay of adaptation. Figure 7*A* shows the non-normalized decay curves, which have different starting adaptation coefficient percentages due to the previous impairment to learning based on noise condition (Fig. 3). To better compare the decay rate of each group, we normalized the decay curves (Fig. 7*B*). Figure 7*A* displays the raw adaptation coefficient, and Figure 7*B* displays the adaptation coefficient as a percentage of the value at the end of training.

Figure 7*B* shows the normalized adaptation coefficient percentage over consecutive error-clamp trials in the decay period (see Materials and Methods). During the decay block, participants were exposed to 80 consecutive EC trials in which lateral movement was restricted, allowing for the adapted movement to return to baseline. To best determine which exponential fit model would best capture the rate of decay, we performed an Akaike information criterion (AIC) test with a single-phase and a two-phase exponential model. Based on the results of the AIC, we determined that a two-phase exponential fit would best capture the rate of decay:

$$y\;=\;Ae^{bx}+Ce^{dx}.$$

This two-phase model assumes that the rate of decrease is the result of a fast $\left(y\;=\;Ae^{bx}\right)$ and slow $\left(y\;=Ce^{dx}\right)$ exponential decay, both of which are concurrent throughout the decay period (Fig. 7*D*). For the fast decay process, the control, 3 N, and 7 N groups had similar decay rates (control: *b*_control _= −0.19, 95% CI [−0.23 −0.14], *R*^2 ^= 0.98; 3 N: *b*_3N _= −0.32 95% CI [−0.38 −0.26], *R*^2 ^= 0.97; 7 N: *b*_7N _= −0.36, 95% CI [−0.45 −0.26], *R*^2 ^= 0.93), though the confidence intervals for the control group decay rates did not overlap those of the 3 N or 7 N groups. For the slow decay process, there were similar decay rates across groups (control: *d*_control _= −0.01, 95% CI [−0.02 −0.005]; 3 N: *d*_3N _= −0.008, 95% CI [−0.01 −0.005]; 7 N: *d*_7N _= −0.004, 95% CI [−0.01 0.002]). Regardless of the varying levels of noise introduced, it is important to note that the 95% confidence intervals for all decay rates overlap.

To better understand how the average adaptation coefficients differed at specific time periods of decay, we examined the average adaptation coefficient as a percentage of the value at the end of training from the first 10% of decay trials (early trials), the middle 10% (middle trials), and last 10% (late trials; Fig. 7*C*,*D*). A two-way ANOVA demonstrated that there was not a significant difference in adaptation between motor noise groups (two-way ANOVA; *F*_(2,35)_ = 0.84; *p* = 0.44; *η*^2^ = 0.01) but there was a significant difference in adaptation during different temporal windows during decay (*F*_(2,35)_ = 102.07; *p* = 2.56 × 10^−13^; *η*^2^ = 0.87), as one would expect. This observation suggests that decay rates following adaptation are relatively resistant to added motor noise.

## Discussion

In this study, we sought to understand the underlying mechanisms and timescales by which externally applied motor noise impairs the initial motor adaptation and the retention and decay of the learning. We hypothesized that this noise impacts the learning rate specifically through the fast learning process of the two-state modeling framework used to capture short-term motor adaptation. To test this, we utilized an established motor adaptation paradigm in which participants made arm reaching movements between two targets and had to adapt to lateral perturbations (a velocity-dependent force-field) in addition to externally applied motor noise. The participants were divided into three separate groups, in which artificial motor noise was applied at either 3 N or 7 N, compared with a control group that did not experience the motor noise. We found that as the magnitude of motor noise increased, the overall learning rate decreased. Applying the two-state model revealed that this overall decrease in adaptation could be explained by impairments to the fast learning process while the slow learning process remained relatively unimpaired. In addition, the applied motor noise had little effect on the retention and decay of adaptation—behavioral aspects that mainly involve the slow learning process.

### Motor noise affects different processes underlying adaptation

The slow learning process has been shown to be largely responsible for both the overall decay and 24 h retention of motor adaptation (Smith et al., 2006; Joiner and Smith, 2008; Wolpert et al., 2011; Albert and Shadmehr, 2018; Sarwary et al., 2018; Alhussein et al., 2019). Thus, based on our hypothesis that noise impacts the learning rate specifically through the fast learning process, we expected to observe no significant differences in the retention of adaptation across levels of motor noise magnitude. While initial findings appeared to suggest that retention was impacted by the motor noise (Fig. 6*A*), similarities in the overall reduction of adaptation over the 1 min delay revealed that the retention was relatively unaffected by increasing motor noise magnitude (Fig. 6*B*). Observing no significant differences in the reduction of adaptation between conditions was also confirmed by the two-state model; the slow learning process (the main component of long-term retention of learning; Joiner and Smith, 2008) was not modulated by motor noise magnitude (Fig. 5).

The fast learning process has previously been shown to quickly respond to movement errors (Smith et al., 2006; Joiner and Smith, 2008; Shadmehr and Mussa-Ivaldi, 2012; Wolpert et al., 2011; Albert and Shadmehr, 2018; Sarwary et al., 2018; Coltman et al., 2019). In theory the more variability in the error, the more impairment there will be in determining the appropriate recalibration of the motor output due to increased uncertainty. Our results suggest that it is the fast learning process that is impaired by the noise added to the force-field perturbation. This increase in uncertainty of the movement disruption may be captured by predictive models, such as the impaired learning rate parameter captured in the two-state model (Fig. 5). Increasing uncertainty can impair the ability of predictive models to capture learning. Increasing noise, a source of uncertainty, lowers the signal-to-noise ratio, which may influence the model to learn less from the error and more from the noise, which we see with the impaired learning curves of the 3 N and 7 N groups (Fig. 3). With noise distracting the modeling process from accurately learning from the error signal, or in the current case, the lateral perturbation, we impair subsequent state estimations from being able to accurately predict and adapt to the error, thus resulting in poorer sensorimotor adaptation over the course of trials. We see this reflected in Figure 3 adaptation coefficient learning curves demonstrating poorer adaptation for high noise groups. As the fast process must quickly respond to changes in sensory information, such as a change in force between the previous and current trial, the model's extraction of fast process learning parameters might learn more from the noise, rather than the lateral perturbation, because the model does not distinguish between these sources of error when making estimations. This could ultimately make it difficult to accurately estimate the current state and lead to impaired learning (the suboptimal behavior as we observe here for the 3 N and 7 N groups).

The current two-state model treats error as a constant, though real-world motor errors are often “noisy” and have diverse characteristics (e.g., temporal, spatial, etc.). Our results demonstrate that increasing the magnitude of motor noise, or rather manipulating a specific characteristic of the noise, impairs motor adaptation by impacting the fast process's temporal sensitivity to the error while the slow learning process remains relatively intact. When faced with noisy error, the fast learning process's contribution may be reduced. Perhaps, this can be due to the fast process having noise-induced gain control and, as an effect, diminishes the slow process. It is clear that early in the learning curve (where the fast process contributes the most to overall adaptation) the initial steep, immediate increase in the learning rate is most reduced as the magnitude of motor noise increases (Fig. 3). According to the feedforward model described in Wolpert et al. (1994), increasing the noise, or uncertainty, of the sensory input will result in a decrease in relying on sensory information to predict optimal motor output. This instead leads to a reliance on the feedforward prediction. As shown in Figure 5, by increasing the noise level of the robotic manipulandum, there is a decrease in the fast learning process, which compared with the slow process is relatively more sensitive to changes in incoming sensory information. Thus, our study provides an initial step into utilizing the two-state framework to understand how underlying mechanisms of adaptation are impacted by noisy motor errors. Overall, our study provides an initial step into utilizing the two-state framework to understand how underlying mechanisms of adaptation are impacted by noisy motor errors. In doing so, we set the foundation to update the two-state model framework's treatment of error as a constant to its more realistic “noisy” presence.

While previous studies (Smith et al., 2006; Joiner and Smith, 2008) have demonstrated the predictive power of the two-state model, there is currently no biological basis for the fast and slow learning processes. McDougle et al. (2015) sought to better understand what mechanisms constitute the fast and slow learning processes and proposed that implicit learning may be closely associated with the slow learning process and explicit learning may approximate the fast learning process. Both the fast learning process and explicit learning occur during the early phase of motor learning. Doyon and Ungerleider (2002) proposed that corticostriatal and corticocerebellar circuits have increased functional activity during the early phase of motor learning, which has been supported by both behavioral and clinical studies (Doyon and Benali, 2005). Indeed, early phases of motor learning, particularly explicit learning through trial-and-error, have been found to be associated with regions of the dorsolateral prefrontal cortex, as well as the cerebellum (Halsband and Lange, 2006). This aligns with previous studies of motor adaptation; online motor corrections and error feedback are largely attributed to contributions from the cerebellum (Wolpert et al., 1998; Bastian, 2006; Tseng et al., 2007; Schlerf et al., 2012; Popa and Ebner, 2019; Tzvi et al., 2022). Regions of the brain that filter noise to allow for goal-directed adaptation appear to overlap, as prefrontal cortex→basal ganglia→thalamic pathways have been demonstrated to help select between relevant and nonrelevant stimuli (Wimmer et al., 2015; Marton et al., 2018; Nakajima et al., 2019; Waschke et al., 2021; Sych et al., 2022). Additionally, the cerebellum has been suggested to play the role of an adaptive filter during online error correction during movement, which involves sorting through sensorimotor noise (Fujita, 1982; Medina and Lisberger, 2007; Requarth and Sawtell, 2011). Thus, the overlap between the brain regions involved in early motor learning and those responsible for filtering sensorimotor noise may play a role in the current results. Specifically, the application of motor noise impairs the fast learning process, which has the most influence during the early phase of adaptation to the velocity-dependent force field. Our behavioral results suggesting that increasing noise specifically impairs the fast learning process implicates that perhaps this could be due to interference with the corticostriatal (Doyon and Ungerleider, 2002; Doyon and Benali, 2005; Wimmer et al., 2015; Marton et al., 2018; Nakajima et al., 2019; Waschke et al., 2021; Sych et al., 2022) and corticocerebellar (Fujita, 1982; Wolpert et al., 1998; Bastian, 2006; Halsband and Lange, 2006; Medina and Lisberger, 2007; Tseng et al., 2007; Requarth and Sawtell, 2011; Schlerf et al., 2012; Popa and Ebner, 2019; Tzvi et al., 2022) circuitry during the initial phase of adaptation. However, these theories are likely a broad interpretation of a complicated network. Further studies examining these relationships are needed to better understand what neural networks are responsible for these different learning processes and the extent these neural mechanisms are also involved in compensating for different noise sources and characteristics.

### Implications for impaired learning in different clinical populations

Tremor—the involuntary, rhythmical movement of the body (Deuschl et al., 2001; Elble, 2017)—is a type of noise that is present during both the preparation of movement as well as its execution. Externally generated tremor is induced when a source outside the organism causes involuntary shaking movements of any part of the body (e.g., an electric toothbrush). Conversely, internally generated tremor is induced when a source from within the organism results in involuntary, oscillatory movements (e.g., the pathological tremor observed in essential tremor, ET). Tremor is an example of noise that can be directly measured in terms of its source, magnitude, and frequency. Similar to other examples of noise, tremor has been shown to impair motor learning and control (Lakie et al., 1995; Kronenbuerger et al., 2007; Shill et al., 2009; Lakie, 2010; Hanajima et al., 2016; Alty et al., 2017; Lopez-de-Ipina et al., 2021; Bindel et al., 2023). For example, Shill et al. (2009) utilized a classical eyeblink conditioning paradigm to compare healthy control participants with ET patients. During the first block of the task, they found that motor learning was impaired by 55.6% in ET patients compared with controls, and this impairment was sustained throughout the entirety of the task. While it is established that tremor-dominant movement disorders have impaired motor learning, the characteristics of tremor and their impact on the underlying components of motor learning, including adaptation, are not well quantified. However, the literature shows that ET frequency ranges between 5 and 10 Hz accompanied by large amplitudes (Louis, 2005; Jankovic, 2008), which we used to inform our noise characteristics. To better understand how variability in internally generated error, such as tremor, impacts patients with movement disorders, we first had to establish how variability in error impacts healthy subjects. This study provides a foundation for understanding how increasing the magnitude of tremor impacts specific underlying mechanisms of motor learning in healthy subjects, demonstrating that varying specific characteristics of noise, such as magnitude of tremor and tremor source, affects motor learning by impairing the fast learning process. If internally generated tremor in disease models plays a role in impairing motor learning, we would expect that it would do so through similar means.

Internally generated tremor, such as the pathological tremor seen in movement disorders, has also been shown to impair motor adaptation. Patients with essential tremor (ET), a neurological condition hallmarked by persistent tremor, are known to have impaired motor adaptation and skill acquisition. Challenges in balance, movement, and postural stability (Rao et al., 2006; Heldman et al., 2011) can lead to difficulties in self-agency and daily living (Lorenz et al., 2006; Anderson et al., 2007). The observed deficits in ET have been suggested to be attributed to the cortico-thalamo-cortical loop in addition to the cerebello-thalamo-cortical pathway (Fasano et al., 2010). The disruption of brain circuits involved in ET are located primarily in the cerebellar cortex (Welton et al., 2021), which is known to govern balance and movement control (Morton and Bastian, 2004). As previously mentioned, increased activity in the cortex, thalamus, and cerebellum is implicated in the early phases of motor learning (and, by association, possibly the fast learning process) as well as the selection of relevant information from sensory noise. It is possible that the same neural mechanisms that are disrupted in ET also contribute to the fast learning process impairment during adaptation to externally generated noise. Deep brain stimulation of the thalamus, which relays sensorimotor information from the cerebral cortex to other motor regions within the brain, has been shown to reduce deficits, such as postural stability and tremor severity in ET patients (Wong et al., 2020). In a study by Bindel et al. (2023), initial motor learning was impaired in ET patients compared with healthy controls, but deadaptation rates between the two groups were similar, suggesting that retention is unimpaired in ET motor learning. These results are aligned with the current findings that motor noise mainly affects the fast learning processes.

A major responsibility of the motor system is determining the source of movement errors to correctly recalibrate motor output (Scheidt et al., 2001; Joiner and Shelhamer, 2006; Berniker and Körding, 2008; Wei and Körding, 2009; Dam et al., 2013; Berniker et al., 2014; Kong et al., 2017). For example, when failing to push open a heavy door, the motor system must determine where the error originated. Is this due to the door itself (is it heavier than assumed?) or is this due to not pushing hard enough (is the exerted force less than assumed?). The motor system relies heavily on integrating information from multiple sensory systems to determine the error source (Ernst, 2006; Körding et al., 2007). It is thought that this is accomplished through reliance on the temporal characteristics of sensory changes to determine if error should be attributed to internally generated or externally based sources. In general, if sensory changes occur gradually over time, the source of error is attributed to the self (Fercho and Baugh, 2014). However, if sensory changes occur quickly, the source of error is often attributed to the external environment (Fercho and Baugh, 2014). After determining the error's source, the motor learning system can adapt/recalibrate motor output to achieve a desired outcome. Thus, if the door remains hard to open after several tries (over time), one might adjust how hard they are pushing on it.

Based on this difference in credit assignment, it is possible that the addition of the externally applied motor noise may interfere with error attribution in a different way than internally generated tremor. Further research is needed in these tremor-dominant movement disorder populations to compare the impact of internally generated tremor in patients versus externally applied motor noise in healthy control. Developing a more comprehensive understanding of the precise aspects of motor learning that are impaired in different movement disorder populations will assist in identifying possible treatment strategies and clinical assessments for specific motor learning deficits (Doyon, 2008; Nieuwboer et al., 2009).

### Possible limitations

With higher levels of motor noise, such as in the 7 N condition, participants must use greater amounts of force to correct their reaching movement. Over time, this use of force may lead to fatigue. In our study, participants were given the opportunity to rest in between experiment blocks, which has been shown to decrease the impact of fatigue on performance (Verhoeven and Newell, 2018). There is also an immediate separation of learning rate performance early on during the training blocks between groups. If fatigue was the sole factor in explaining our results, it is likely we would largely see the largest difference and/or a decrease in learning rate during the later trials during training. The observation that this was not the case suggests that it is unlikely that fatigue significantly contributed to the observed differences between groups.

Additionally, based on our experiments, we can conclude that the applied noise impaired the ability to learn the required temporal pattern of force, but we cannot determine directly if this was the result of cocontraction. During FF trials, it is likely participants cocontracted to counteract the lateral perturbation (Franklin et al., 2003; Milner and Franklin, 2005). However, we relied on measurements from subsequent EC trials (which did not contain the lateral perturbation) to quantify the ability to adapt to previous FF trials. It is unclear if participants continued this cocontraction during EC trials without the presence of the perturbation, as muscle activation patterns tend to differ in changing environments (Osu et al., 2003). As the purpose of our current study was to determine the extent participants compensated to the standard movement perturbation [i.e., the force-field (FF) perturbation] with added levels of applied noise, we aim to investigate this possibility in future experiments.

In our study, we used the applied motor noise to examine the possible effects of externally generated tremor on motor learning. However, it is difficult to extrapolate these findings directly to other noise sources (e.g., internally generated tremor due to brain disorders). Thus, even though it provides valuable insight into how the nervous system handles increasing noise during adaptation to error, we should note that the externally applied motor noise in the current study may have limitations in the generality to patient populations. For example, it is currently unclear how physiologically relevant 3 N and 7 N of noise magnitude compares to pathologic tremor (Elble, 1986; Calzetti et al., 1987; Elble et al., 2006; Deuschl et al., 2022). However, through our findings, we can now understand that when magnitude of noise increases, ability to adapt decreases, which can then be tested in the context of ET. The frequency (10 Hz), which was standard across conditions, is most closely related to the tremor frequency experienced by patients with ET, whose tremor tends to range from 5 to 10 Hz (Louis, 2005; Jankovic, 2008). Overall, we aimed to model the general manifestation of a single characteristic of pathological tremor, which is typically described as high amplitude (Elble, 1986; Calzetti et al., 1987). Through this experiment, we provide preliminary evidence that increasing the magnitude of externally generated tremor affects motor adaptation specifically by impairing the fast learning process. Future studies will aim to investigate how changing other characteristics of tremor, such as frequency and source, influence specific underlying characteristics of motor learning and control.

## Synthesis

Reviewing Editor: David Schoppik, New York University Grossman School of Medicine Decisions are customarily a result of the Reviewing Editor and the peer reviewers coming together and discussing their recommendations until a consensus is reached. When revisions are invited, a fact-based synthesis statement explaining their decision and outlining what is needed to prepare a revision will be listed below. The following reviewer(s) agreed to reveal their identity: Michael Landy.

Major concerns:

1. The nature of the added noise and the motivation for the force levels and frequency are unclear. Based on Figure 1b, it appears that the noise applied forces only along the x-axis (i.e., perpendicular to the primary axis of reaching movements), but this is not clearly explained in the Method. The Discussion makes it seem as though the experiments were trying to simulate Essential Tremor (ET), but this is not mentioned in the introduction. As stated, a 10 Hz frequency is within the range of ET tremor frequencies, but the manuscript should clarify how or why the two force magnitudes were chosen.

2. The manuscript details tests of reaches in two directions and appears to have combined all reaches for analysis. However, it is not clear that this was justified. A figure (either in the main text or as a supplement) that depicts (1) exemplar trajectories from each of the three groups in the baseline and training phases, and (2) demonstrates that the overall kinematics were comparable across movement directions within each group would clarify this choice.

3. Figures 2a and 4a show the group average force profiles across the baseline and training trials, respectively. They appear to be centered at the point of peak velocity (although this is not clarified anywhere), but it is not clear which axis is plotted. Is it the force in the x-axis? If so, then the "ideal force" profiles in Figure 2a are confusing. They appear to correspond to those that would counter a velocity-dependent force field. Yet, according to the paragraph beginning on line 205, the baseline trials were conducted in a null field. In this case, the "ideal force" profile should be flat, like the "actual force" profiles for the three subject groups. See also line 950, which prompted us to wonder again what the average force profile is. Is it a single number? A function of time within the trial? From the plots, it's a scalar, which makes it odd to call it a "profile." In the bottom row of Figure 4: The three plots should really all have the same scales, despite needing to contain the outlier in the left-hand one.

4. We were surprised by how little between-subject variance there is in the force profiles for the 3N and 7N groups in Figures 2a and 4a, especially in comparison to the control group. We would expect the sudden unpredictable lateral perturbations resulting from the added noise to induce some rapid feedback correction behavior (i.e., participants would reflexively push back against the force). The variance shown by the 3N and 7N groups in Figure 2b suggests this is the case in the baseline phase, but the variance in the force profiles in Figure 2a does not depict this. Can the authors confirm that the force profiles plotted depict the correct error bars?

5. The criteria for valid reach trials are unclear. Lines 172-174 specify that a successful movement to the target was achieved when the peak movement velocity was within a range of 0.25-0.35 m/s, and the movement duration was less than or equal to 800 ms. Lines 287-290 further state that trials that did not meet these speed and duration requirements were discarded, and this affected a fairly large percentage of trials for some groups. Yet, almost none of the movement times shown in Figure 2C are below 800 ms, and a number of the subject means for peak velocity shown in Figure 2D exceed 0.35 m/s. Further, the text does not state whether participants were given feedback about unsuccessful reaches, what kind of feedback, and what instructions they were given.

6. Throughout the paper, the manuscript reports significant main effects and/or interactions resulting from ANOVA. In almost every case, the results involved comparisons over 3 or more means. Yet, there are no reported post hoc analyses on these effects. Instead, the manuscript simply states the means and standard error values and interprets the results based on "eyeballing" the differences. This is not in keeping with the requirements for statistical analysis in papers published in eNeuro and likely produced interpretation errors as there is no accounting for variance in the distributions producing each mean, which was substantial in many cases (e.g., the control group in Figures 3b and 4b). Post hoc means comparisons would accurately break down the significant omnibus ANOVA results.

7. The problem noted in the previous comment is particularly apparent when interpreting the results of the data analysis shown in Figure 6. The manuscript reports a significant interaction between the factors of group and trial (Figure 6a). Instead of conducting post hoc tests to determine what is causing the interaction, the adaptation coefficient delta values are compared across the groups in a second ANOVA (Figure 6b). This latter analysis produces no significant main effect, which conflicts with the interaction found in the former analysis. In the Discussion, the interpretation is that there is no difference in retention across groups. Yet, in examining Figure 6b, the control group distribution appears to have much higher variance than the other group distributions and even appears bimodal. This suggests that the lack of significant difference in delta values could have resulted from a messy control group rather than a lack of effect of added noise.

8. Lines 474-483: The manuscript reports the results of analysis comparing the decay rates of a two-phase exponential model fit to their data. The conclusion that there is no difference in the decay rates, but this is based solely on the results from the slow process. There is a clear difference in the parameter values for the fast decay process --- the CI for the control group does not overlap with that of the 3N and 7N groups. This must be included when interpreting their results. Additionally, to supplement the model-based analysis, we suggest including an analysis comparing the behavior (shown in Figure 7b) at different time points similar to that done for the training phase behavior.

9. The Discussion could be much more focused. The current text skims existing literature on the two-state learning model, without really commenting on how this study fits into it. It also includes a lengthy discussion of Essential Tremor, which is then undercut by the finding that the physiologic relevance of the added noise to ET is unclear.

10. How constrained are the estimates of the parameters by the data in the two process model? Please consider a cross-validation or bootstrap analysis to provide confidence intervals for the parameter estimates.

Line-by-line major concerns.

188: The middle term is meant to be a 2x2 matrix and should be laid out that way. More to the point, the sine wave is incorrectly stated (line 202 is missing the "sin()" the sum is not put into an equation. Please do so.

205: Does "null movement" mean no noise or also no force field?

206-207: "experienced noise" is true during some trials, but not during familiarization trials.

213: 100+20 = 120!

237-238: "by linearly regressing the baseline-subtracted actual force": This is unclear. It reads like this refers to a scalar value (per trial), but force is a function of time. At this point the manuscript should state whether it refers to peak force (before or after the baseline subtraction) or average or whatever it is.

255: Again, this is a scalar for each trial, but what does "net motor output" mean if it's a scalar?

261: All these equations are about force. But force error is not what is sensed but, rather, perceived positional error. Also, the sign is backwards here (if f(n)+x(n) is zero, then one has correctly cancelled the external force).

274-275: "the weight of the internal state": Your equations for the model just states that the two processes are summed. It doesn't indicate a weighted sum. So, what's this about?

276-280: This is unclear: "The amount of error on the current trial...was normalized as either f[n]=0". f[n] is not the error; it's the perturbation. "When f[n]=0 during EC trials" is also unclear. During EC trials, f = -x by definition, since movement is clamped.

360 or so: The manuscript doesn't provide any kind of significance test of the rates or the asymptotes (or their differences across groups). See also Major comment #6.

451-454: The manuscript analyzes and plots differences here. But, in analogy to the "gain" of adaptation, wouldn't it make more sense to compute and test the change as a proportion, not as a difference?

468: The AIC is computed based on log likelihoods, but the manuscript does not discuss the noise model and thus how you would compute the likelihoods that go into the AIC.

522: Less sensitivity of the slow process to errors in the environment? The manuscript is unclear here, since the process exists to correct an error. Perhaps it is more that the fast process has a noise-induced gain control? We are unclear what model the authors have in mind here and the description is too vague.

Fig. 1 legend: What direction was 90 vs 270 force?

917: The 10 Hz is applied force, which is not usually described as oscillatory movements as they are not the perturbation, but rather one of its consequences along with stiffening the arm.

922: "participants were asked to hold..." only on some of the trials, not all

Minor Concerns:

1. According to Figure 1c, the Baseline phase of the task comprised 120 trials with 20 error clamp (EC) trials randomly interspersed, but the text specifies the Baseline phase containing 100 trials with 10 EC trials interspersed (lines 207-210).

2. Figure 1b depicts five movement types, but the figure caption describes only four. The article text (lines 175-176) also only describes four movement types (that differ from those described in the Figure caption).

3. The y-axis label for Figure 2E is clipped.

4. The paragraph beginning on line 410 appears out of order. The first sentence references "results above (i.e., similar retention and decay rates across the difference subject groups)," which are actually presented in the following two sections of the results beginning on lines 438 and 459, respectively.

5. Figure 7 and its description in the text and caption appear to contain errors. Figure 7a appears to show the "raw" adaptation coefficient, but the y-axis in the figure has it as a percentage. Panels b and c appear to display the adaptation coefficient as a percentage of the value at the end of training, but this should be clarified in the text and caption.

6. Typographical or grammatical errors:

Line 439: insert "to which" between "extent" and "retention."

Line 444: "Largely" is not the appropriate word here; consider replacing it with "relative."

Line 477: the subscript on the b for the 3N group is mislabeled.
